# Template-Aware Transformer for Person Reidentification

**DOI:** 10.1155/2022/8917964

**Published:** 2022-04-01

**Authors:** Yanwei Zheng, Zengrui Zhao, Xiaowei Yu, Dongxiao Yu

**Affiliations:** ^1^School of Computer Science and Technology, Shandong University, Qingdao 266237, China; ^2^Department of Mathematics and Statistics, Jiangsu Normal University, Xuzhou 221116, China

## Abstract

Person reidentification (ReID) is a challenging computer vision task for identifying or verifying one or more persons when the faces are not available. In ReID, the indistinguishable background usually affects the model's perception of the foreground, which reduces the performance of ReID. Generally, the background of the same camera is similar, whereas that of different cameras is quite different. Based on this finding, we propose a template-aware transformer (TAT) method which can learn intersample indistinguishable features by introducing a learnable template for the transformer structure to cut down the model's attention to regions of the image with low discrimination, including backgrounds and occlusions. In the multiheaded attention module of the encoder, this template directs template-aware attention to indistinguishable features of the image and gradually increases the attention to distinguishable features as the encoder block deepens. We also increase the number of templates using side information considering the characteristics of ReID tasks to adapt the model to backgrounds that vary significantly with different camera IDs. Finally, we demonstrate the validity of our theories using various public data sets and achieve competitive results via a quantitative evaluation.

## 1. Introduction

Face recognition (FR) is widely used to identify or verify one or more persons in the scene using a stored database of faces. However, the face is not available in the case of security cameras, or the number of face pixels is very small, which cannot be used for FR. Person reidentification (ReID) recognizes pedestrians using apparent information across camera views at different locations and times [1], which avoids the requirement that the faces must be front and close-up. ReID is regarded as a subproblem of image retrieval and is mainly used in the field of public security. ReID faces a significant challenge due to changes in people's poses, camera viewpoint, occlusion, etc. Due to its wide application and great academic challenges, ReID has become a hot research field. With the development of the convolutional neural network (CNN), ReID has made great progress in recent years [2, 3].

Luo et al. [4] have revealed that the effective receptive field of CNN is smaller than the theoretically expected one, which indicates that it lacks the ability to capture surrounding context information. Transformer [5], however, does a better job, as it can establish long-range dependencies using attention mechanisms. As a result, it recently has been developed rapidly in computer vision. Vision transformer (ViT) [6], which is the first pure transformer network applied to image recognition, divides the image into blocks and feeds them into the encoder to obtain the image feature representation. TransReID [7] proposes the jigsaw patch module (JPM) and embeds side information including camera and viewpoint into the ReID task. It has also made significant progress. By utilizing the attention information between different image patches, the transformer has effectively improved the global receptive field.

**Figure 1:**
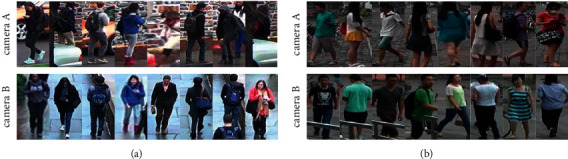
Illustration about the correlation between background style and camera ID. Images with the same camera ID are highly similar in their background, while those with different camera IDs differ significantly. (a) DukeMTMC-reID. (b) Market-1501.

Nevertheless, there are still some issues unsolved with the ReID task. (1) Transformer focuses on self-attention within the sample, rather than mutual attention between samples. Since ReID is a retrieval task, which is essentially a task of comparing image similarity, the mutual attention for intersamples can help make better discrimination for different persons. (2) Typically, a ReID task employs a small number of fixed cameras, resulting in similarities in the background with poor discrimination [8], as Figure 1 shows. Most existing methods pay too much attention to the relevance between appearances of images [9], rather than to get precise foreground features and omit background features [10]. As a result, a few background patterns appear in large numbers, which introduces noise into the model learning process, lowering its accuracy. (3) Although side information such as camera and viewpoint can enhance feature robustness in ReID [11, 12], the CNN-based method of fusing side information is still unsuitable for the transformer. To minimize the bias of side information variations, it is necessary to redesign a specific module to construct an invariant feature space [7].

To address the aforementioned issues, we propose a template module for learning similar features (so-called indistinguishable features) among samples and improving the perception of distinguishable features. We integrate it into the attention stage of the transformer and construct a template-aware transformer (TAT). Specifically, we design a learnable template that is in the same shape as the flattened patches of the image after linear projection. We then concatenate the template with the input image embedding. After the position embedding is added, we feed it into the transformer encoder. In the attention stage, in addition to computing self-attention, we also compute the attention between the image patch and the template patch (i.e., template-aware attention). It assists in matching indistinguishable features of images, such as reducing the negative consequence of background and occlusion. Since the backgrounds from a certain camera are similar in style and those from different cameras vary widely, we introduce the camera as side information and assign different templates to different cameras. This template module used by TAT is simple to set up and can be easily integrated into the transformer and applied to other image retrieval tasks.

The main contributions of this paper are summarized as follows:

1. We propose a learnable template module for learning indistinguishable features among samples and improving its interest on distinguishable features
2. Aiming at the characteristics of ReID tasks, we introduce camera ID as side information to better expand the template and have effectively improved the model in its ability to discriminate input samples from different cameras
3. Extensive experiments show this model outperforms other state-of-the-art methods on Market-1501 [13], DukeMTMC-reID [14], and Occlude-Duke [15].

The remainder of this article is organized as follows: section 2 discusses the related work about the proposal and development of transformer and presents related work of person reidentification. In Section 3, the template-aware transformer is presented, including an agent (a learnable template) and its expansion way.  Section 4 introduces some details of the implementation and provides the experimental results.  Section 5 concludes this article and outlines the future work.

## 2. Related Work

This work is closely related to visual transformer models and ReID methods, especially those related to saliency, attention, and alignment. In this section, we will briefly discuss these efforts.

### 2.1. Person Reidentification

ReID usually consists of two steps, feature representation and feature matching. Since 2014, deep models, especially CNN, have been widely used for ReID to enhance both these two steps. At the early stage, deep learning methods based on global features are the preferred approaches. To obtain fine-grained features, methods using local feature representation learning are proposed. These two representation learning methods are often combined for ReID tasks. Researchers have proposed feature matching methods based on stripe [16–18] and grid patch [19, 20]. Moreover, multichannel and multiscale methods [21–23] are also used to capture local features.

Pursuing more robust feature representations, the auxiliary information is introduced into the training, such as viewpoint information [24], camera information [25, 26], timing information [27, 28], and data augmentation [29]. Many studies [23, 30] have also modified the design of the backbone network to better implement the characteristics of ReID. Some researches use heuristic methods to enhance the performance of classification [31–33]. In addition, unsupervised learning methods [34–36] for ReID have also been studied intensively in order to better implement in real-world applications.

### 2.2. Visual Transformer

Transformer [5] was proposed in natural language processing (NLP) tasks. It aims at building encoders and decoders using attention mechanisms. Subsequently, the transformer has been applied in vision tasks such as target detection [37–39] and semantic segmentation [40, 41]. Recently, in the field of image classification, ViT [6] has applied pure transformer structures to nonoverlapping image patches.

In order to decrease the amount of computation of ViT, researchers have proposed many methods. Touvron et al. [42] introduce a teacher-student strategy for knowledge distillation, which can reduce ViT's reliance on large amounts of pretrained data. Wang et al. [43] introduce the pyramid structure into ViT, making it better at doing dense prediction as a backbone. Liu et al. [44] use the sliding window method to reduce the computation amount of transformer self-attention.

### 2.3. Person Reidentification on Transformer

Some researches on person ReID introduce a transformer into the existing CNN architecture. For instance, Zhang et al. [45] integrate transformer architecture into CNN and take advantage of both CNN and transformer for person ReID, Li et al. [46] use the transformer encoder-decoder architecture to implement occluded ReID in a unified deep learning model, and Ma et al. [47] combine local part features with an attention mechanism. To overcome the shortcomings of CNN in context-awareness and the loss of detailed information caused by convolution and downsampling, He et al. [7] propose a pure transformer-based object ReID framework and are strongly competitive with CNN-based approaches. Zhu et al. [48] propose an auto-aligned transformer that adaptively locates human parts and nonhuman parts to extract local part features.

### 2.4. Attention Mechanism for ReID

The attention mechanism is used in many computer vision problems [49]. Early researchers typically use simple alignment methods to mitigate the effect of background on human recognition. Zheng et al. [50] propose a pedestrian alignment network (PAN) which self-adaptively locates and aligns pedestrians within the bounding box using the attention mechanism of CNN. Guo et al. [51] design two branches to solve the human part misalignment and nonhuman part misalignment problems, where the branches focus on the human part and latent part, respectively. Some saliency-based methods [52–56] have also been proposed for mining different salient features to obtain different clues of pedestrians.

In order to obtain finer divisions, researchers have conducted a lot of research in semantic segmentation based on attention mechanisms. Tian et al. [9] propose a deep human parsing network for background-foreground separation and by setting random backgrounds to do data augmentation. Song et al. [57] use a fully convolutional network (FCN) [58] for semantic segmentation to obtain human mask, separate human body and background, and then learn features separately from body and background regions to eliminate the influence of background on identification. Cai et al. [59] design two attention modules based on the JPPNet [60] semantic segmentation network for filtering the influence of background and extracting global and local features. Considering that the JPPNet model cannot generate limb masks accurately, Huang et al. [61] design SBSGAN to generate soft-mask images and mitigate background rather than completely remove it to reduce domain gaps. To enhance the focus on the discriminative parts of the input scene, Ding et al. [62] use a feature mask network to automatically learn global and local features specific to the identity of certain target persons. However, most of these methods are based on CNNs and are not suitable for direct application to transformer structures. Thus, we still need better designs to enhance the discriminative ability of the transformer for ReID.

## 3. Methodology

### 3.1. Overall Architecture

Various network architectures have been introduced to learn the feature representation of ReID. To have a better visual contextual association, we use the ViT architecture to extract features on person ReID tasks. Additional modules built on this architecture are designed to improve its performance.

**Figure 2:**
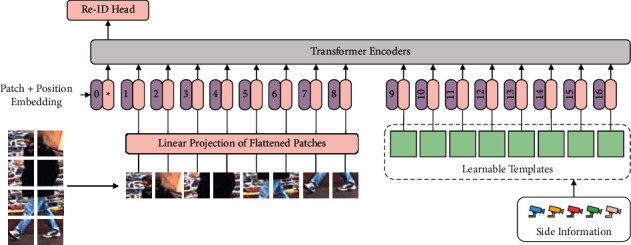
Pipeline of proposed TAT. A set of learnable template embeddings, together with a class token (CLS) and flattened patches, are designed as inputs to the transformer encoder. The side information of the input image is used to select the appropriate template.

Figure 2 depicts the overall architecture of the proposed method. During the training phase, the provided input image is sliced into several same-sized blocks and transformed into flattened patches by linear projection (e.g., convolution and flattening operations). A class token (CLS) is attached in front of the flattened patches. A sequence of position embeddings is summed with the patches to discriminate the relationship between the different patches in their position. The prepared embeddings are fed into transformer encoders for encoding operations. Our approach appends an additional set of embeddings, called learnable templates, after the CLS and the flattened patches. The position embedding is also expanded and summed. Then, the embeddings mentioned above are fed into the encoders together.

The encoders of the transformer are made up of multiple encoding layers. In each encoding layer, attention is computed between each part of the incoming embeddings. After multiheaded self-attention, multilayer perceptron, and normalization operations, the new embeddings are output from this layer and served as input to the next layer. In the final layer, only the first embedding (i.e., CLS) in the output is treated as the feature representation of this image and is used for classification and loss calculation, while the other embeddings are discarded. In the training phase, back propagation optimizes the parameters of the entire network as well as the proposed learnable templates.

### 3.2. Learnable Template

The self-attention mechanism of the transformer is to capture the context information of a single image. If we turn to the mutual attention of two images, such a mechanism is excruciatingly time-consuming. For image retrieval tasks like ReID, there is a similarity computation process between different images. If we apply the attention mechanism to similarity calculation (e.g., when we input two images into a transformer), we must compute mutual attention pairwise between query and gallery datasets. The time complexity is *O*(*N*_*q*_ × *N*_*g*_), where *N*_*q*_ and *N*_*g*_ are the numbers of images in query set and gallery set, respectively. In contrast, the time complexity of the traditional method is *O*(*N*_*q*_+*N*_*g*_). Thus, it can be seen that mutual attention is not reasonable, whether on dataset-based learning or actual applications, since it significantly increases the time spent on the ReID validation process. As an alternative, we design an agent, which is a learnable template, to avoid excessive time spent like this while increasing the interaction between different image attentions.

The standard ViT [6] model transforms the input image from *x* ∈ *ℝ*^*H*×*W*×*C*^ to 2D flattened embedding *x*_*p*_ ∈ *ℝ*^*N*×(*P*^2^ · *C*)^, where (*H*, *W*) is the resolution of the input image, *C* is the number of channels, (*H*, *W*) is the resolution of the patch, and *N*=*HW*/*P*^2^ is the number of patches. The learnable embedding, class token *x*_*cls*_, is prepended to the patch sequence and used as the image representation. The sequence input to the transformer encoder can then be obtained as follows:

(1) $$\begin{array}{c} Z_{0}=\left[x_{\text{cls}};F\left(x_{p}^{\left(1\right)}\right);F\left(x_{p}^{\left(2\right)}\right);\ldots ;F\left(x_{p}^{\left(N\right)}\right)\right]+P, \end{array}$$

where *𝒵*_0_, *𝒫* ∈ *ℝ*^(1+*N*)×*D*^, ℱ is the linear projection function, *x*_*p*_^(*i*)^ is the *i*^*th*^ term of *x*_*p*_, *𝒫* is a learnable position embedding, and *D* is the dimension of each embedding.

We design a set of learnable template vectors in the same shape as the flattened embeddings of the image and concatenate them to *z*_0_, as Figure 2 shows, resulting in a new input sequence.

(2) $$\begin{array}{c} {\tilde{Z}}_{0}=\left[x_{cls};F\left(x_{p}^{\left(1\right)}\right);F\left(x_{p}^{\left(2\right)}\right);\ldots ;F\left(x_{p}^{\left(N\right)}\right);T^{\left(1\right)};T^{\left(2\right)};\ldots ;T^{\left(N\right)}\right]+\tilde{P}, \end{array}$$

where ${\tilde{𝒵}}_{0},\tilde{𝒫}\in {\mathbb{R}}^{\left(1+2N\right)\times D},T^{\left(i\right)}$ is the *i*^*th*^ term of template *𝒯* ∈ *ℝ*^*N*×*D*^, and $\tilde{𝒫}$ is the expanded position embedding.

In the training stage, each image will first use the template to predict its own classification and then calculate the loss function and update the template by back propagation. The template functions as a kind of bridge, enabling indirect interactions between images. Since all images share the same template in the prediction process, the template will gradually learn to represent the common features, that is, indistinguishable features, of all images.

### 3.3. Template-Aware Attention

Each transformer encoder consists of multiheaded self-attention (MSA) and multilayer perceptron (MLP). A layernorm (LN) and a residual connection are set separately before and after the MSA and MLP.

(3) $$\begin{array}{c} {\tilde{Z}}_{\ell }^{\prime }=\text{MSA}\left(\text{LN}\left({\tilde{Z}}_{\ell -1}\right)\right)+{\tilde{Z}}_{\ell -1},\;\ell =1,\ldots ,L,\\ {\tilde{Z}}_{\ell }=\text{MLP}\left(\text{LN}\left({\tilde{Z}}_{\ell }^{\prime }\right)\right)+{\tilde{Z}}_{\ell }^{\prime },\;\ell =1,\ldots ,L, \end{array}$$

where *L* is the number of encoder layers.

The self-attention module in MSA computes the response sequences at each position by estimating the attention scores and determining how much focus to place on other positions. The computation of attention scores is based on query, key, and value vectors, which are derived from the layer normed input vector. We compute the dot product with a set of key vectors for each query vector, then normalize, and translate them into probabilities with softmax to obtain the attention weights. The weights are applied to the value vectors to get the final attention.

(4) $$\begin{array}{c} \left[Q,K,V\right]=ZU_{QKV},\\ \text{Attention}\left(Q,K,V\right)=\text{Softmax}\left(\frac{Q\cdot K^{⊤}}{\sqrt{d_{k}}}\right)\cdot V, \end{array}$$

where **Q**, **K**, **V** ∈ *ℝ*^(1+*N*)×*D*^ are the query, key, and value vectors, respectively, which are generated by multiplying the input sequence against three learned metrics *U*_*QKV*_ ∈ *ℝ*^*D*×*d*_*k*_^, and $\sqrt{d_{k}}$ is used for normalization. Multiple self-attention heads are computed in parallel.

We introduce the concept of template-aware attention for the attention module. It is based on the learnable template, allowing the transformer to focus on the area of samples that are unrelated to classification, such as background or occlusion. We compute the attention scores between different input vectors in the MSA and normalize them to *S*_*n*_.

(5) $$\begin{array}{c} S_{n}=\frac{\left(\left[\begin{array}{c} Q_{x_{p}}\\ Q_{T} \end{array}\right]\cdot \left[\begin{array}{cc} K_{x_{p}}^{⊤} & K_{T}^{⊤} \end{array}\right]\right)}{\sqrt{d_{k}}}\\ =\frac{1}{\sqrt{d_{k}}}\left[\begin{array}{cc} Q_{x_{p}}K_{x_{p}}^{⊤} & Q_{x_{p}}K_{T}^{⊤}\\ Q_{T}K_{x_{p}}^{⊤} & Q_{T}K_{T}^{⊤} \end{array}\right]. \end{array}$$

Then, the final attention is calculated.

(6) $$\begin{array}{c} \text{Attention}=\text{Softmax}\left(S_{n}\right)\left[\begin{array}{c} V_{x_{p}}\\ V_{T} \end{array}\right], \end{array}$$

where the vectors derived from the input *i* are packed into three different matrices, namely, query vector **Q**_*i*_, key vector **K**_*i*_, and value vector **V**_*i*_.

**Figure 3:**
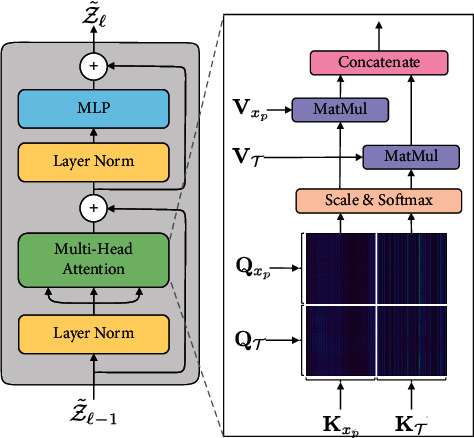
The calculation of single-head attention (ignoring cls token). **Q**_*x*_*p*__, **Q**_*𝒯*_ and **K**_*x*_*p*__, **K**_*𝒯*_ are used to do matrix multiplication to get the four parts of the attention map.

In addition to computing the self-attention map between image patches, as shown in equation (5) and Figure 3, we also need to compute the template-aware attention map between image and template, as well as the self-attention map of the template itself.

In the shallow encoder block, the **Q**, **K**, and **V** of image and template can be clearly distinguished in terms of function. Image patches focus more on distinguishable features, whereas template patches focus more on indistinguishable features. As the encoder blocks deepen, the two kinds of features will gradually merge, and both focus on the samples' distinguishable features.

### 3.4. Template Expansion

Despite the fact that the template has learned some indistinguishable features and diminished the concern about them using template-aware attention, the template's information capacity is limited by its size. Due to the large amount of data and the scene-bias problem, it is difficult to collect, learn, and distinguish all the indistinguishable features of the entire dataset with such a scale of the template. However, arbitrarily increasing the size of the template increases the time complexity linearly, which is not reasonable. To solve this problem, we propose a method of expanding the template based on side information.

It is envisaged that the role of templates in the training process is to learn the similarities between samples. Therefore, it is reasonable to use the same template for similar backgrounds and different templates for those with significant different backgrounds. Here, we define the expand operation as simply increasing the number of templates to *N*_*c*_, where *N*_*c*_ is the number of cameras, and obtain the expanded template *𝒯*_*c*_ ∈ *ℝ*^*N*_*c*_×*N*×*D*^. For images with camera ID *r* ∈ [1, *N*_*c*_], we assign the *r*^*th*^ template *𝒯*_*c*_[*r*] as the corresponding template. During the training process, each template *𝒯*_*c*_[*r*] can learn the indistinguishable features of the images with camera ID *r*. Since the images with the same camera ID have a large area of overlapping or similar background, the indistinguishable features learned by *𝒯*_*c*_[*r*] will be very representative.

### 3.5. Training Objective

In the training phase, we use the common cross-entropy loss and triplet loss to train the model. The output of the cls token ${\tilde{𝒵}}_{L}^{\left(0\right)}$ in the last layer represents the feature of the input image, and the other outputs ${\tilde{𝒵}}_{L}^{\left(1:2N\right)}$ are discarded. The cross-entropy loss is calculated as follows:

(7) $$\begin{array}{c} L_{cls}\left(x,y\right)=-\log\frac{e^{x_{y}}}{\left(\sum_{c=1}^{C}e^{x_{c}}\right)}, \end{array}$$

where *x*_*i*_ is the probability that the image is predicted to be of class *i*, *y* is the target, and *C* is the number of classes.

The triplet loss is calculated as follows:

(8) $$\begin{array}{c} L_{\text{tri}}={\left[d_{p}-d_{n}+\alpha \right]}_{+}, \end{array}$$

where *d*_*p*_ and *d*_*n*_ denote the Euclidean distances between the anchor and positive/negative sample features, respectively, and hyperparameter *α* controls the margin of loss.

The following is the final objective function of our model:

(9) $$\begin{array}{c} L=L_{\text{cls}}+L_{\text{tri}}. \end{array}$$

## 4. Experiments

In this section, we describe the experimental details and validate the effectiveness of the proposed TAT on several widely used holistic datasets and an occluded dataset.

### 4.1. Datasets

We performed experiments with our model on four datasets, Market-1501 [13], DukeMTMC-reID [14], Occlude-Duke [15], and MSMT17 [63]. The detailed information about each dataset is given in Table 1. One camera ID indicates the same background in each dataset.

  Market-1501 uses a total of 6 cameras, containing 5 high-resolution cameras and 1 low-resolution camera. It captures 32,668 pedestrian image bounding boxes of 1,501 identities using Deformable Part Model detection. 750 identities are used for training, and 751 identities are used for testing. Each person has an average of 3.6 images per viewpoint. Each annotated identity presents in at least two cameras so that a cross-camera search can be performed. For testing, 3,368 query images were used as the query set to match among 19,732 gallery images with 2,793 distractors.
  DukeMTMC-reID consists of 36,411 images acquired from 8 different cameras and provided with manually annotated bounding boxes. 1,404 identities appear in more than two cameras, and 408 distractor identities appear in only one camera. 16,522 images with 702 IDs are randomly selected as the training set, while the remaining 2,228 query images with 702 IDs and 17,661 gallery images with 702 IDs as well as 408 IDs (as distractors) are assigned as the test set. One query image for each ID in each camera is picked into the test set, and the remaining images are put in the gallery set.
  Occluded-Duke is a dataset on occluded scenes, which is created by filtering from the DukeMTMC-reID dataset. It contains 15,618 training images, 17,661 gallery images, and 2,210 query images. All query images have occlusions (e.g., trees, cars, and other people) in varying degrees, while the gallery set contains both holistic and occluded images.
  MSMT17 is a large multiscene multitime dataset that is close to real scenes. It contains totaling 126,441 bounding boxes of 4,101 identities captured by 12 outdoor cameras and 3 indoor cameras, which are captured under different weather and lighting conditions. The images of the dataset are randomly divided according to the training-test 1 : 3 ratio, that is, the training set contains 1,041 identities with a total of 32,621 bounding boxes, while the test set includes 3,060 identities with a total of 93,820 bounding boxes.

### 4.2. Implementation Details

#### 4.2.1. Model Architectures

We use the base size of ViT model proposed by Alexey et al. [6] as the backbone network. The model contains 12 transformer encoder layers and 12-headed attention, with hidden size *D*=768, normalization parameter *d*_*k*_=8, and multilayer perceptron (MLP) size 3072. We also introduce those useful tricks proposed by He et al. [7], including overlapping patches, jigsaw patch module (JPM), and side information embeddings (SIE).

#### 4.2.2. Training Details

The input image is resized to 384 × 128 and sliced into overlapping patches with size 16 × 16 and stride 12 × 12. For data augmentation, we use horizontal flipping, random cropping, and random erasing [29]. The transformer backbone is initialized with pretrained parameters on ImageNet [64]. The batch size is set to 64, and each ID has 4 images in a mini-batch. Triplet loss margin *α* is set to 0. We use the SGD optimizer with 0.9 momentum and 1e-4 weight decay. The training stage is conducted for 120 epochs, and the cosine decay learning rate is set with an initial value 0.032. In the early stage of training, a linear warmup is used to grow the learning rate. We set warmup_step=1000. Our model is implemented using the PyTorch framework and is experimented on 4 NVIDIA GeForce RTX 3090 GPUs.

#### 4.2.3. Evaluation Metrics

For evaluation, we adopt standard metrics, namely, cumulative matching characteristic (CMC) curves and mean average precision (mAP). All experiments are run under the single query setting, and Rank-1 results are reported. To be consistent with most other studies, postprocessing methods such as reranking [65] are not used in the evaluation phase.

### 4.3. Comparison with State-of-the-Art Methods

We compared our result with some state-of-the-art methods on three widely used holistic benchmarks and one occluded benchmark, as shown in Table 2.

#### 4.3.1. Results on Holistic Datasets

**Figure 4:**
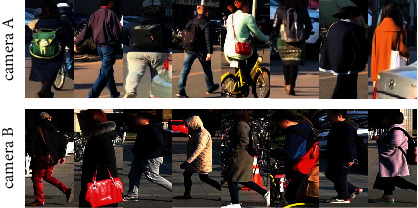
Overview of images from the MSMT17 dataset. With the same camera ID, the backgrounds are variable and lack indistinguishable features over large areas.

Our method performs well on Market-1501 and DukeMTMC-reID. Since the performance on these two datasets is almost saturated, the mAP of TAT is very close to the mAP of those state-of-the-art methods, but its Rank-1 outperforms the previous methods by 0.4% and 0.8%, respectively. Yet on MSMT17, our method performs worse, which may be related to the overly complex background of this dataset. As shown in Figure 4, there is no clear pattern exhibited by the backgrounds of MSMT17, such as the same material of walls, floor tiles, plants, and frequently photographed occlusions. Therefore, our method is not appropriate for this dataset. Furthermore, this result also demonstrates that our method is specifically designed for indistinguishable features.

#### 4.3.2. Results on Occluded Datasets

The occlusions in the occluded dataset are usually the same or similar objects. TAT adapts better to the indistinguishable features and thus should perform better on the occluded dataset. Experiments show that our method yields excellent results on the occluded dataset Occluded-Duke, outperforming other state-of-the-art methods by 1.4%/1.2% for mAP/Rank-1. Compared with the holistic dataset, the performance improves more significantly on the occluded dataset, which agrees with the above conjecture.

### 4.4. Ablation Study of Template

From Table 3, we see a 0.2%/0.3% improvement in mAP on the two datasets with the addition of a single template. The improvement is even more pronounced when the template is expanded by introducing side information like camera ID, resulting in a 1.0%/1.1% improvement in mAP, respectively. We also introduced the mINP metric [2], which is used to evaluate the cost of finding the hardest match, to see the boosting effect of the template. The best result boosts mINP by 2.0%/1.5% compared to the baseline. These experimental results demonstrate the effectiveness of our proposed template structure, where the introduction of side information especially amplifies the performance of the template structure greatly in the experimental results.

The training time increases due to the introduction of the template structure. On these two datasets, the introduction of the template increases the training time from 1.97 h/2.43 h to 3.83 h/4.69 h, respectively. According to our analysis, the template increased the number of parameters of multiheaded attention to approximately two times the original number. Therefore, the increase in training time is basically positively correlated with the increase in the number of parameters.

### 4.5. Ablation Study of Parameter Initialization

The learnable embeddings in deep networks are usually sensitive to the initialization of parameters. A fine initialization of parameters can well improve the results. Take position embedding as an example, people usually initialize it using parameters pretrained on datasets with an enormous number of images like ImageNet-21k. However, it is difficult to pretrain on a huge dataset for our self-defined learnable embedding module.

To investigate the impact of different strategies for initializing the templates, we conduct some experiments on the DukeMTMC-reID dataset. In the experiments, three different initialization strategies are introduced as follows: (i) zero initialization, (ii) truncated normal distribution initialization, and (iii) patch initialization which use the flattened patches of any person image in the dataset as the templates' initialization parameter, as follows:

(10) $$\begin{array}{c} T_{\text{init}}^{\left(i\right)}={\text{Any}}_{x_{p}}\left(F\left(x_{p}^{\left(i\right)}\right)\right). \end{array}$$

We introduce the third strategy mainly for the reason that there is something in common in the meaning of templates and images. The flattened patches of an image are representations of the whole image, while templates are representations of indistinguishable features. Patch initialization may possibly avoid learning the template from scratch while not spending additional pretraining time.

The experimental results are shown in Table 4. The results of the three initialization strategies are close to each other. But compared with zero initialization and truncated normal distribution initialization, patch initialization still gives a stable improvement to the model. On both mAP and Rank-1, patch initialization improves the performance by 0.12% to 0.30%.

**Figure 5:**
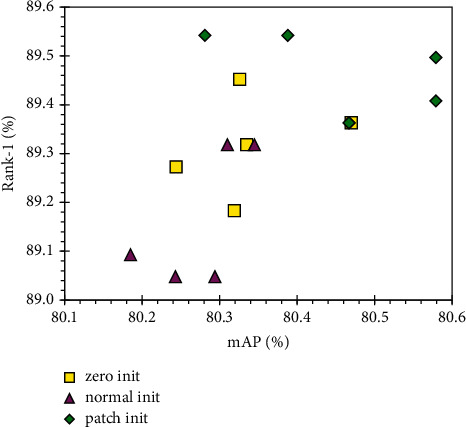
Scatter plot of the results of three different initialization strategies on the DukeMTMC-reID dataset. Each strategy is experimented five times.

Figure 5 shows the scatter plot of the experimental results. According to the scatter plot, patch initialization results are the best, followed by zero initialization, while normal initialization is the worst. It agrees with the conclusion drawn from Table 4. Therefore, the proposed patch initialization strategy is effective in boosting the model performance.

### 4.6. Visualization Analysis

**Figure 6:**
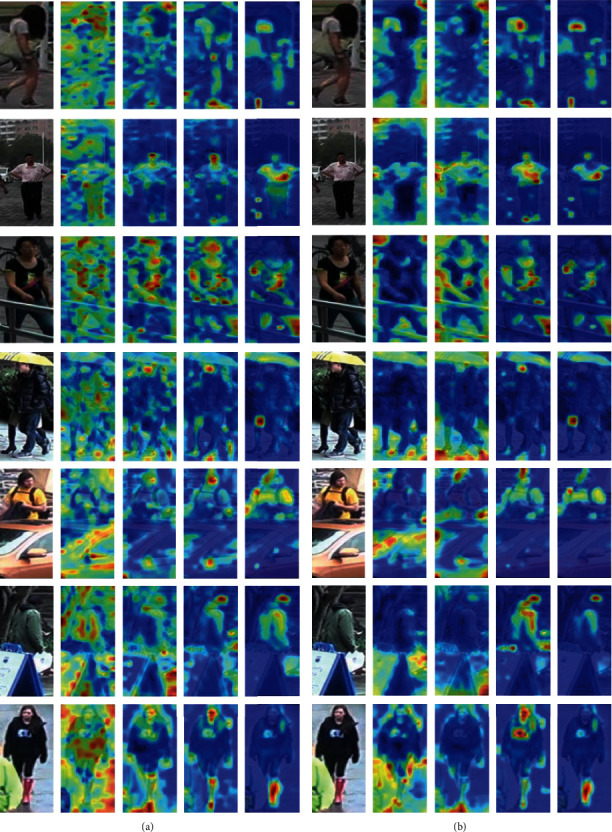
The figures show the mean attention maps of several transformer layers from shallow (leftmost) to deep (rightmost). The attention is indicated from low to high with the color blue to red. How much attention the image pays to each region of itself is illustrated in (a). How much attention the template pays to each region of the image is illustrated in (b). (a) Self-attention. (b) Template-aware attention.

In order to observe the mechanism of the learnable template, we visualized the attention maps of the intermediate outputs of TAT during network inference, as shown in Figure 6. It is observed that the self-attention region of the image itself is gradually transferred from the entire image at the shallower layers to the human body region at the deeper layers. But the template-aware attention works in a different way. While in the deeper layers of the transformer, template-aware attention focuses on almost the same areas as self-attention, namely, the human body parts, and in the shallower layers, however, it focuses on areas outside the human and surrounds the body. Particularly, in the last few images of Figure 6, template-aware attention shows a strong focus on occlusions such as cars, signages, and people.

The visualizations show that in the shallower layers, template-aware attention extracts invalid information pieces, like backgrounds and occlusions, and discards them in the deeper layers. Thus, the model is allowed to focus on people eventually. For this reason, TAT improves more significantly on the occluded dataset than on other datasets, since it filters out large areas of occlusion information in the image.

## 5. Conclusion

In this paper, we propose a learnable template that can adaptively learn the indistinguishable features of images. This module can improve the learning ability of the transformer by using template-aware attention. To expand the template, we also introduce side information which enhances the template's adaptability to different scenes. Extensive experiments show that template-aware transformer (TAT) built using these methods outperform many state-of-the-art methods.

From this study, we can also draw a useful conclusion for the ReID problem—the learnable template is effective when there are only a few background patterns, but the number of each pattern is large. The template can learn to focus on the different parts of the image pair. By this way, the discrimination between persons is enhanced in the few background pattern scene. However, the shortcoming of this method lies in the fact that it is not applicable to variable backgrounds. In practical applications, the method is suitable for scenes with a few fixed cameras. It is not recommended to use this method if each camera has only a few pictures.

This is a study on ReID, a popular ranking problem. One of the remaining questions is whether these conclusions can be generalized to other problems, for example, classification, detection, generation. In the later scenes, the backgrounds are different, and the foregrounds are very similar for the same objects. It becomes an interesting topic to ask whether the template can learn to focus on the object parts for the image groups (the same class images), which deserves further and comprehensive study.

## Figures and Tables

**Table 1:** Details of Re-ID datasets.

Dataset	#ID	#Train	#Test	#Image	#Cam
Market-1501	1,501	751	750	32,668	6
DukeMTMC-reID	1,404	702	702	36,411	8
Occluded-duke	1,404	702	519	35,489	8
MSMT17	4,101	1,041	3,060	126,441	15

**Table 2:** Comparison with the state-of-the-art CNN-based and transformer-based methods on different datasets. DukeMTMC refers to the DukeMTMC-ReID dataset. The first group contains CNN-based methods, and the second group contains transformer-related methods.

Methods	Market-1501		DukeMTMC		Occluded-duke		MSMT17	
	mAP	*R* − 1	mAP	*R* − 1	mAP	*R* − 1	mAP	*R* − 1
PGFA [15]	76.8	91.2	65.5	82.6	37.3	51.4	—	—
PCB + RPP [66]	81.6	93.8	69.2	83.3	—	—	40.4	68.2
*P* ^2^-Net [51]	85.6	95.2	73.1	86.5	—	—	—	—
OSNet [23]	84.9	94.8	73.5	88.6	—	—	52.9	78.7
HOReID [67]	84.9	94.2	75.6	86.9	43.8	55.1	—	—
MGN [68]	86.9	95.7	78.4	88.7	—	—	52.1	76.9
BAT-net [69]	87.4	95.1	77.3	87.7	—	—	56.8	79.5
ISP [70]	88.6	95.3	80.0	89.6	52.3	62.8	—	—

Pirt [47]	86.3	94.1	77.6	88.9	50.9	60.0	—	—
PAT [46]	88.0	95.4	78.2	88.8	53.6	64.5	—	—
AAformer [48]	87.7	95.4	80.0	90.1	58.2	67.0	63.2	83.6
TransReID [7]	89.5	95.2	**82.6**	90.7	59.2	66.4	**69.4**	**86.2**

ViT-baseline	86.5	94.2	79.3	88.9	53.1	60.5	61.0	81.8
TAT (ours)	**89.7**	**95.8**	82.5	**91.5**	**60.6**	**68.2**	59.1	80.5

**Table 3:** The effectiveness of the template and its expansion. *𝒯* denotes the template, while *𝒯*_*c*_ denotes the templates expanded using camera information.

Methods	Market-1501			DukeMTMC-reID		
	mAP	*R* − 1	mINP	mAP	*R* − 1	mINP
ViT-baseline	86.5	94.2	62.8	79.3	88.9	45.2
+ *𝒯*	86.7	94.1	63.4	79.6	89.2	45.4
+ *T*_*c*_	**87.5**	**94.5**	**64.8**	**80.4**	**89.3**	**46.7**

**Table 4:** The performance and standard deviation on the DukeMTMC-reID dataset with three different initialization strategies.

Init strategy	mAP	*R* − 1	mINP
Zero	80.34 ± 0.08	89.32 ± 0.10	46.89 ± 0.16
Normal	80.28 ± 0.06	89.17 ± 0.14	46.91 ± 0.14
Patch	**80.46** ± 0.13	**89.47** ± 0.08	**46.97** ± 0.30

## Data Availability

The datasets we used in this paper are all publicly available. We have cited the datasets in the manuscript. Because the datasets are published by other researches, we do not list the URL here. The readers can find the citations in the references. We will release the source code at https://github.com/template-aware/TAT after the paper is published.
